# Challenges and Facilitators for HIV Testing Services and HIV Self-Testing Programming During Emergency Care in Kenya: A Qualitative Study of Patients

**DOI:** 10.63620/mkjshar.2024.1002

**Published:** 2024-01-15

**Authors:** Scarlett J Bergam, Josephine Chen, John Kinuthia, John Wamutitu Maina, Sankei Pirirei, Rose Bosire, David Bukusi, Harriet Waweru, Daniel K Ojuka, David A Katz, Carey Farquhar, Michael J Mello, Adam R Aluisio

**Affiliations:** 1 George Washington University School of Medicine and Health Sciences, Washington DC, USA; 2 Brown University, Providence, USA; 3 Department of Research & Programs, Kenyatta National Hospital, Nairobi, Kenya; 4 Department of Accident and Emergency, Kenyatta National Hospital, Nairobi, Kenya; 5 Center for Public Health Research, Kenya Medical Research Institute, Nairobi, Kenya; 6 HIV Prevention Unit, Kenyatta National Hospital, Nairobi, Kenya; 7 Department of Surgery, University of Nairobi Faculty of Health Sciences, Nairobi, Kenya; 8 Department of Global Health, University of Washington, Seattle, USA; 9 Department of Epidemiology, University of Washington, Seattle, USA; 10 Department of Medicine, University of Washington, Seattle, USA; 11 Department of Emergency Medicine, Alpert Medical School of Brown University, Providence, USA

**Keywords:** HIV Testing, HIV Self-Testing, Kenya, Emergency Care, Injury, Qualitative Data

## Abstract

**Background:**

Young people in Sub-Saharan Africa, especially young males, have been insufficiently engaged in HIV Testing Services (HTS). In Kenya, these persons are often treated in emergency departments (EDs) for injuries, a healthcare interaction that could be leveraged for HTS including HIV self-testing (HIVST). There is however, limited data from patients on ED-HTS which impedes programmatic advancement.

**Methods:**

A qualitative study was completed to understand challenges and facilitators for ED-HTS and HIVST delivery in Kenya (12/2021-03/2022). Data were collected via 28 in-depth patient interviews (14 males, 14 females) who had been treated for injury in the Kenyatta National Hospital (KNH) ED. Transcripts were coded, summarized, and analyzed with Dedoose^™^ software with both inductive and deductive codes to captured a priori and emergent themes. Data were mapped to the Capability-Opportunity-Motivation Behavioral (COM-B) model for health behavior change to identify interventions which could be used to enhanced ED-HIV services delivery.

**Results:**

Themes for challenges for ED-HTS included lack of testing, stigma, incompatibility with patient condition and lack of perceived confidentiality. Patients identified a range of facilitators for ED-HTS such as health maintenance, convenience, relevance to ED setting and trust in healthcare providers. Challenges to ED-HIVST programming included perceived testing inaccuracy, psychological impact and difficulties with follow-up. Themes on facilitators included greater privacy, supportive autonomy, time efficiency, increased accessibility and ease of testing. In mapping the data to the COM-B model, main identified themes for capability were adequate resources access and promotion of patient perceptions on testing importance. Opportunity themes identified appropriate timing for ED-HTS engagement and autonomy with HIVST use. Data themes for motivation included correct understanding of testing results, supportive patient-provider interactions, and increased agency in testing choices with varying options. These data correlated to intervention functions of education, environmental restructuring, enablement, training, modeling, incentivization an-persuasion which could be used to develop appropriate programs to promote ED-HTS delivery.

**Conclusion:**

ED patients view HTS and including HIVST as favorable. Although challenges exist, multiple pragmatic interventions can be leveraged in ED-based HIV services to enhance program development and impacts to deliver testing too difficult to reach persons already in contact with health systems.

## Introduction

There are nearly 38 million people living with HIV (PLHIV) globally: 70% reside in sub-Saharan Africa [1]. HIV accounts for more than a million fatalities, and 10% of disability-adjusted life years lost, annually [2–4]. Injuries are another widespread global health issue affecting approximately one billion people and causing nearly five million mortalities per year [5]. Like HIV, injuries primarily burden lower- and middle-income countries (LMICs), where ninety percent of all injury-related mortalities occur [6, 7].

Persons suffering injuries in sub-Saharan Africa are also at high risk for HIV. Across SSA, the HIV-seroprevalence in injured patients is significantly greater than matched national disease burdens [8–10]. Populations at an elevated risk for injury in sub-Saharan Africa include children and adolescents, refugees, urban populations, sex workers and transgender persons [11–15]. In Kenya, young men are most likely to be injured, and simultaneously account for the majority of undiagnosed PLHIV, having the lowest HIV testing engagement [16–21]. Based on these findings, in Kenya advisory bodies have identified young adults and males as focus priority groups for improved HIV testing, a goal which could be addressed during healthcare interactions for injuries [19–22].

In Kenya injury care is primarily obtained in emergency department (ED) settings, which is a care period during which patients have been shown to be receptive to health advancement [23]. Although HIV testing for high-risk persons is recommended during health encounters in high prevalence countries such as Kenya, delivery in SSA EDs has not been well achieved [24]. Commonly identified challenges to ED-based HIV Testing Services (HTS) include poor infrastructure, stigma and care time constraints [25–28]. In addition, ED-based HTS in injured patients may be overlooked due to being considered unrelated to the acute presentation [29]. These factors can perpetuate low testing and missed case finding in high-risk patients already in contact with health services.

HIV self-test (HIVST) delivery is an approach in ED-HTS programming that may have benefits as an adjunctive option [29–32]. HIVST kits can address some challenges pertaining to resources, as well as patient empowerment and confidentiality [33]. HIVST programming is a recommended strategy to improve testing for young adults and males, making the ED injury population a target for this modality [34]. While HIVST has not been broadly implemented in Kenyan EDs, research demonstrates positive impacts among young adults and males from other healthcare contexts in SSA [35]. In Kenya, there is limited data from ED settings, and specifically from injured persons, on HTS and HIVST programming. This lack of information inhibits the development and implementation of effective approaches for HIV testing and care delivery among high-risk emergency care patients already in contact with health systems.

## Methods

This formative qualitative study elucidates challenges and facilitators pertaining to ED-based HTS and HIVST programming in Kenya. Qualitative data was collected using semi-structured research agendas utilized in-depth interviews (IDIs). Agendas were developed to address the following qualitative research questions: (1) challenges of HTS within the ED, (2) facilitators to HTS within the ED, (3) challenges to ED-HIVST programming, (4) facilitators to ED-HIVST programming. The study site was Kenyatta National Hospital (KNH), the largest public healthcare facility in Kenya. The KNH ED has HTS available at all times staffed by voluntary counseling and testing (VCT) providers and follows national reporting documentation and procedures (i.e., anyone diagnosed with HIV is offered follow-up and linkage to care for treatment) [36]. There is access to free HIVST kits based on supply availability from the ED service delivery point [37]. The study was approved by the KNH ethics and research committee (P29/01/202) and the Rhode Island Hospital Institutional Review Board (1501033-3).

### Data Collection

The semi-structured research agenda for the IDI was developed and refined based on prior literature and through an iterative approach by study investigators with content and qualitative methods expertise in HIV research and care delivery in Kenya. Data were collected between December 2021 and May 2022. Data collection was primarily carried out in person in confidential settings at the KNH hospital campus. Two IDIs were completed via phone due to participants being unable to come to the study site secondary to their health status.

Audio recorded qualitative data sessions were conducted in English and/or Kiswahili (based on participant preference) by a male Kenyan bilingual qualitative facilitator with training in qualitative methods and attended by a female Kenyan bilingual notetaker. Research personnel had no prior relationship with participants. Audio data was de-identified, translated into English, and transcribed. Transcripts were reviewed during post-sessions debriefs during the data collection phase by study personnel to identify both a priori and emergent data, as well as address any gaps in the agendas to improve data collection.

### In-Depth Interviews

The IDI study sample was composed of patient stakeholders. IDIs were chosen for data collection among patients to support comfort and confidentiality in discussing the participants’ potentially sensitive experiences and perceptions on HTS. Adult patients (>18 years of age) who had been treated in the ED for injuries and able to provide informed consent were eligible for inclusion. Patients known to be pregnant, prisoners of the state, and those unable to provide informed consent were excluded. Injury designation was based on the standardized triage classification used in the study setting [38]. Eligible participants were screened for enrollment at ED disposition and informed consent was completed prior to their data collection session. Purposive sampling was used to populate participants in the IDIs to yield a representative stratified sample of males and females who were exposed or were not exposed to HTS during their emergent health encounter. IDI lasted 60-90 minutes; a setting-appropriate reimbursement was provided. Transcripts were reviewed by the study team continuously and enrollment was completed once data saturation had been achieved. Consensus on data saturation was reached at 28 IDIs.

### Data Management & Analysis

Qualitative data were imported for coding and analysis into Dedoose^™^ software version 4.3 (Manhattan Beach, USA). The transcripts were coded using a parallel inductive and deductive approach which allowed for capture of both a priori and emergent themes. All transcripts were coded independently by two trained study personnel. Coders were trained with study team evaluation of six transcripts in which data were compared and codes discussed to ensure intercoder reliability (concordance >85.0%) and achieve consistent master codes across analyses. Applied, thematic analysis was used to ensure comprehensive identification of key concepts [39]. As such, the formalized approach to the coding process was iteratively applied until no new emergent themes and related codes were identified. From the thematic analysis cross-cutting themes on 1) challenges and facilitators for ED-based HTS and 2) challenges and facilitators specific to HIVST provision in the ED setting were identified. The Capability-Opportunity-Motivation Behavioral Model (COM-B), which examines health behavior through these interrelated lenses, was utilized as a framework to overlay the key findings of the analysis and identify intervention functions which could be used to promote behavioral health change tp enhance ED-based HTS inclusive of HIVST [40].

## Results

Of the 28 IDI participants, 14 had completed HIV testing during their index encounter for emergent care and 14 had not been tested. Half of the sample was female (n=14) and half was male (n=14). One-third (n=9) of the sample was < 25 years old (Table 1).

### Challenges for Emergency Department HIV Testing Services

Patients reported-challenges focused on lack of awareness of and perceived importance for testing, incompatibility of testing with patient condition, stigma, confidentiality and logistical resource aspects.

- Patients lacked knowledge about the availability, purpose, and benefits of ED-HTS: “I wasn’t aware about things like that. I wasn’t aware” (IDI6, Male, >25, Not Tested). As one patient said: “Because I was not raped, I could not imagine that I could be told such” (IDI7, Female, <25, Not Tested) referring to ED-based HTS. Another patient stated: “…I was tested six months ago, so I feel okay” (IDI 2, Male, <25, Not Tested).
- The condition for which the patient was in the ED was viewed as a potential challenge by patients, as well as illustrated by this patient quote: “First of all, my head was not in good shape. Then, I had a fracture, I was not able to sit, so they didn’t do any test then” (IDI 20, Male, >25, Not Tested). Other patients stated: “My intentions in the hospital were not to be tested for VCT” (IDI 6, Male, >25, Not Tested).
- Patients identified a concern that their confidentiality regarding HIV status would be threatened by HTS when they presented at the ED for injury. One participant reported: “I was at the clinic and I heard one of the medical practitioners saying, "look at that big man showing off, we tested and found him positive. You know that is a person who cannot maintain ethics” (IDI 20, Male, >25, Not Tested). Another participant stated that the ED is not an appropriate setting for HTS: “They should have admitted me first and then taken me to a room where I can stay for a while to familiarize with the environment and then later go and get tested” (IDI 19, Male, >25, Tested).
- Patient participants perceived staffing as a challenge to ED-HTS, just one aspect of resource factors raised by participants: “They should have doctors specifically for HIV testing” (IDI 28, Male, <25, Not Tested).
- Patients expressed their fear of a positive test and its psychological effects on their well-being: “There is someone who has said that when I get tested, I will start thinking about many things and die. That is, when you test positive, you will have a lot of thoughts on your head, and I didn’t want such thoughts” (IDI 5, Female, >25, Tested).

### Facilitators for Emergency Department-HIV Testing Services

Patients offered a range of facilitators for ED-HTS. These included perceiving testing as a form of regular health maintenance, positive patient-provider relationships, expanded engagement for comprehensive ED-HTS, means to protect health care providers, and efficient use of ED care encounters.

- Six patients stated that ED-HTS is a way to maintain health “You get to know your status, how you are health-wise” (IDI 17, Male, >25, Tested).
- Three patients stated that a strong facilitator for testing was the relevance of the HTS to their reason for presentation: “It was a good thing because when we found the accident, blood flowed, so I didn’t know if I had contracted the disease. So, it was good to know my status early” (IDI 4, Female, <25, Tested).
- Patients report that having trustworthy providers was a facilitator for successful ED-HTS: “These guys are reliable […] they can’t give you false results” (IDI 3, Male, <25, Not Tested).
- Patients identified that they would be open to ED-HTS interactions in general and from an expanded representation of providers. One patient stated: “I cannot choose whom to test me, because that person is doing their job. For me, I would just want the testing” (IDI 16, Female, >25, Not Tested).
- A patient, who was tested while waiting for care in the ED highlighted this use of time and noted: “A doctor came to talk to me as I waited for my surgery, and I went to some room with a written letter for referral, where I was tested [and] found to be well.” (IDI 3, Male, <25, Tested).
- Two patients discussed their motivation to protect healthcare workers: “People in the ward assist each other and, in the process, your blood may mix with their s and from that, you can get infected with HIV/AIDS” (IDI 19, Male, >25, Tested).

### Challenges to Emergency Department HIVST Programming

Challenges to ED-HIVST programming, included: concerns around proper use of assays, test kit accuracy, psychological risks, and barriers to follow-up.

- Select patient participants reported a lack of confidence in their ability to self-test and understand their results: “I would have used it with the service provider so that he can show me how to use it, because I am not a doctor. I can do something stupid, then it gives me a wrong result or it fails to work” (IDI 19, Male, >25 Tested).
- Five patients also reported a distrust of the accuracy of HIVST: “The one for saliva did not please me because you… you don’t trust it for sure” (IDI 23, Male, >25, Tested).
- From the patient perspective, a reactive HIVST without proper counseling and support from a healthcare provider could have psychological consequences. A patient shared: “You find other people might even climb a building and commit suicide because […] the disease has found them, so they prefer to die” (IDI 5, Female, >25, Tested).
- Seven patients identified concerns about follow-up and linkage to care with HIVST programming: “The only issues I feel there is with self-testing is someone can test with it from home then they find out they are positive and since it says you should go back to a facility for confirmation many people can choose not to go and have it confirmed, they decide to keep it to themselves and end up not starting ART” (IDI 21, Female, <25, Not Tested).
- Two participants doubted patients’ medical and psychological capabilities to choose their desired test while in the ED: “It has its demerits because someone is in pain and going to ask them to choose what they want might not work” (IDI 20, Male, >25, Not Tested).
- Four patients stated that the lack of accountability with HIVST can lead to monetary losses: “You gave out 50 kits. Then at the end of the day, you only get 10 responses back, so 40 kits are unaccounted for, meaning they were either discarded, or not used… The hospital ends up [with] losses” (IDI 21, Female, <25, Not Tested).
- Five patients expressed their fears of disclosing their status due to HIV stigma: “You know if you don’t have courage to test, it can break your marriage” (IDI 13, Male, >25, Tested).

### Facilitators of Emergency Department HIVST Programming

Patients identified facilitators of HIVST programming and use across the themes of improved privacy, supportive autonomy, less invasive serologic assay and temporal efficiency.

- The ability to use a HIVST test in a private location was seen as a benefit among nine patient participants, with one noting: “I think it enhances privacy since not everyone knows you are taking an HIV test. It’s not the same as entering a room [with a sign saying] “VCT” (IDI 21, Female, <25, Not Tested). “…I can lock myself at a room and know my results then throw it away” (IDI 3, Male, 25, Tested).
- The autonomy provided via HIVST kits was viewed as a facilitator by patients. A patient stated: “Its advantage is… there are some people even if you take them to the doctor, they don’t want to be counseled, they just want to get tested, know their status, then they go. It will still help some people.” [IDI 2, Male, <25, Not Tested]. Eight patients stated that the flexibility of the HIVST allows them to meet their goal of testing for HIV regularly: “If the test is given voluntarily or bought, I can buy four and test four times after three to five months” (IDI 13, Male, >25, Tested).
- An appealing characteristic identified for HIVST use was that the assays can be saliva based. A patient stated: “Using saliva is a good alternative because one gets scared when the injection is made” (IDI 5, Male, >25, Tested). Four patients identified this method as more time-efficient than traditional tests: “Patients can prefer the one for swabbing because it’s faster” (IDI 19, Male, >25, Tested).
- Patients identified the time efficiency derived from HIVST kits as a facilitator to programming and use. A patient identified that the ability to test quickly outside of a healthcare setting was a facilitator: “sometimes you find yourself with someone and maybe we want to engage in sex, and we don’t have the time to go to the hospital or we are not in a position to go to the hospital we can use this kit anytime anywhere” (IDI 18, Female, >25, Not Tested).
- Patients discussed the idea of using technology, such as a mobile app, to facilitate HIVST follow-up. One patient said, “An app will be good because one can access details even if they are at a far place. On the other side, some people can lack access to data bundles or a smart phone” (IDI 10, Female, >25, Not Tested).

### Capability-Opportunity-Motivation Behavioral Model to Inform ED-HTS with HIVST Programming

Table 2 maps the data on challenges and facilitators for HTS and HIVST to the COM-B model. The qualitative data is thematically organized across the domains of capability, opportunity and motivation. These organized domains are linked to intervention functions with associated categories of evidence-based behavior change techniques that can be used to address challenges and leverage facilitators to improve ED-based HTS and HIVST delivery.

For the domain of capability, which refers to an individual’s physical and psychological capacity to engage in ED-based HTS, the key themes for physical capability were access to physical testing resources and sufficient adaptive personnel. Psychological capability centered on the perceived importance of HTS amongst patients. Within the study context, opportunity refers to the factors affecting testing access based on the physical and social environment. For social opportunity, primary themes included experiences with previous testing and motivation for testing. Physical opportunity included the availability, timing, administration, and resources for ED-HTS in the context of patient experiences. Motivation refers to the automatic and reflective processes that affect patients’ desire and ability to engage in the health behaviors to achieve HIV testing. Automatic and reflective motivation for ED-HTS is depicted by healthcare workers’ drive to deliver HTS while supporting patients’ perceptions of the positive impacts of HTS. These identified themes correlated to intervention functions of education, environmental restructuring, enablement, training, modeling, incentivization and persuasion which can be acted upon through behavior change techniques inclusive of shaping knowledge, feedback and monitoring, repetition, social support and goal planning (Table 2). [40]

## Discussion

In sub-Saharan Africa ED-HTS, and associated HIVST programming, has not been well studied or leveraged to meet help meet global targets. The current work uses qualitative data and evidence-based frameworks to inform understanding approaches on ED HIV testing. In this study comprising of patients from an emergency care setting in Kenya, multiple challenges and facilitators to HTS and HIVST programming were identified as were potential intervention functions to inform services delivery.

The current results suggest that both facilities-based HTS and HIVST for persons appropriate for self-testing are desired by patients and can be used to reach at-risk persons during emergency care [40]. The mixed experiences and preferences of patients regarding HIV testing in and out of the ED supports a differentiated service delivery approach [41]. There is no single solution for ED-based HIV services however, expanding options to multiple opportunities to engage in testing, such as making HTS and HIVST available at different time points and locations, can help to overcome identified barriers such as fear of confidentiality and discomfort with healthcare providers. This can allow for a greater reach of key and under-tested populations, especially young people and men who often present for injury care.

Patient perspectives on HIVST reveal a variety of impressions and preferences for HIVST. First, HIVST must be paired with support and information, or else there is potential for incorrect use, disposal, or lack of access to follow-up care [42]. Whether HIVST is distributed along with a VCT counselor phone contact, an informational pamphlet, or a mobile app, this informational and social support is essential to overcome the many perceived challenges of HIVST related to understanding testing procedures and results and following up. Furthermore, the psychosocial aspect of ED testing should be considered. Mental health disorders have been associated with other HIV risk factors, such as drug misuse and sexually risky behaviors [43]. Therefore, to combat concerns of negative reactions to a positive test, some prefer taking the test in the presence of healthcare providers to be immediately linked to care. Others stated that they preferred to take it in the comfort of their own homes to process their results emotionally before taking next steps. Either way, patients should be linked to both medical and psychological care before, during, and after testing.

Mapping the data to the COM-B model revealed the multilevel influences across the capability opportunity and motivation domains which impact patents in engaging in ED-HTS and HIVST programming. HIVST gives patients agency and empowerment over the timing, setting, and follow-up of their testing. While this agency comes with potential negative consequences—particularly psychosocial challenges and low perceived accuracy of tests participants suggested ways to roll out ED-HIVST that could increase patient uptake in the ED. These data highlight how education to shape knowledge can be leveraged to strength end user comprehension and ability in HIVST programming. Most preferred in-person HTS approaches in the ED setting, and in the use of HIV self-testing in-person follow-up for reactive tests and telehealth follow-up for non-reactive self-tests. With these vary testing options environmental restructuring, enablement and modeling intervention functions can be used for behavior change techniques focusing on identify and social supports. Patients identified challenges in HIV testing on psychosocial concerns and for HIVST use on fears of lack of access to follow-up care. These can be addressed via programming that uses interventions on enablement and modeling for behavior change focused on associations, self-belief and social support. Mobile health tools were identified as an approach to promote both education and follow up. Specifically, for HIVST programming mobile health applications or the use of an existing messaging service, such as WhatsApp^™^, to support intervention functions in education and enablement could be used to improve understanding of testing and subsequent linkage to care. Prior studies have shown successful mobile health interventions related to HIVST and models of smartphone applications that have facilitated self- and partner-testing in LMICs [44–47]. Smart phone applications and HIVST phone support lines can be used as a model to design and test feasible HIVST mobile health use in the ED setting as an environmental restructuring and enablement intervention [48].

The current data should be interpreted in light of some limitations. This study was conducted in a large public hospital, with existing HTS and an overall population that has testing available in that setting. As not all emergency care facilities have these capabilities, especially in less urban areas, the results may not be as generalizable to those settings [48]. Due to the COVID pandemic and physical disability in some participants, two patient interviews were conducted by phone. However, no thematic differences were noted based on the interview modalities used. Finally, this study did not specifically ask patient stakeholders about specific intervention functions or approaches to improve HTS programming. Therefore, the suggested approaches derived from the data must be further assessed in clinical application.

## Conclusion

The data from this study provide challenges and facilitators from patients in Kenya on ED-based HTS including HIVST distribution and uses the COM-B model to identify data-driven approaches which could enhance testing programming. As emergency care venues represent an innovative environment to improve testing for high-risk persons already in contact with health services, further study on the topic and evaluation of the pragmatic approaches are needed and have the potential to help contribute to advancing global HIV control efforts.

## Figures and Tables

**Table 1:** Characteristics of Participants

Characteristic	N (%)
**Sex**	
Male	14 (50.0%)
Female	14 (50.0%)
**Completed HIV Testing During ED Care**	
Tested	14 (50.0%)
Not Tested	14 (50.0%)
**Age (years)**	
<= 25 Years	9 (32.1%)
>25 Years	19 (67.9%)
**Relationship Status**	
Married / In a relationship	14 (50.0%)
Single	11 (39.3%)
Divorced / Widowed	2 (7.1%)
Wishes not to disclose	1 (3.6%)
**Highest Educational Attainment**	
Primary schooling or less	8 (28.6%)
Secondary schooling	12 (42.9%)
Diploma / Certificate	5 (17.9%)
Bachelor’s level or higher	3 (10.7%)
**Employment Status**	
Full-time laborer	9 (32.1%)
Full-time professional	2 (7.1%)
Self-Employed	9 (32.1%)
Not working / Other	8 (28.6%)
**Established Primary Care Provider**	
No	14 (50.0%)
Yes	14 (50.0%)
**Self-Reported Condom Use**	
Always	3 (10.7%)
Sometimes	8 (28.6%)
Never	15 (53.6%)
Wishes not to disclose	2 (7.1%)

**Table 2:** Application of the COM-B Model of Behavior Change to Inform HIV Testing Services

COM-B Component	Themes	Illustrate Quotes		Intervention Functions	Behavior Change Techniques
		Facilities-Based HIV Testing	HIVST Programming		
Physical Capability	Resource Access Personnel Availability	“The best time to get tested at the A&E—they may not deal with the HIV first, but your emergency first, then test you later. But for one who is not very sick, they can get tested first” (IDI 20, Female, >25, Not Tested)	“They can put those two options, so those that are not in much pain might prefer the one for drawing blood samples, but those in a lot of Pain, you know they cannot help themselves, they can go to…even parents can prefer the other one, the one for swabbing because it’s faster. And also, a lot of people can get tested from that” (IDI 19, Male, >25, Not Tested)	Education Environmental Restructuring Enablement	Shaping Knowledge Feedback & Monitoring Antecedent
Psychological Capability	Perceived Importance	“Before treatment, I was in pain, so HIV testing would not be important to me. Maybe if I was admitted, I would be tested” (IDI 7, Female, <25, Tested)	“Imagine telling the doctor on yourself that you are positive, like “doctor, I am positive” that is impossible. Like I don’t have the courage” (IDI 9, Male, <25, Not Tested)	Training Environmental Restructuring	Identity Self-belief Social Support
Social Opportunity	Prior testing experiences Drive for testing services	“Let me say I was always scared of the results. I remember the first time I was so scared I even center the room first and came back later for the results. It wasn’t a good experience, it was very scary” (IDI 18, Female, >25, Not Tested)	“Yeah, the patient can actually pick the kit and test in privacy so that it doesn’t have many eyes looking at it and knowing the results at home and bring it back the following day psychologically prepared, remember many people are not fit psychologically meaning they are mentally sensitive.” (IDI 24, Male, >25, Not Tested)	Modeling Incentivization	Social Support Comparison of Behavior Identity
Physical Opportunity	Time allocation Resource Availability	“I think it’s good after you are received. They take all tests, including your HIV test, so that when they start treating you, they know all the problems you have” (IDI18, Female, >25, Not Tested)	“It’s a good idea since someone can do it even from the washroom, do your test and get your result. If it’s positive you can go back to the person that gave you and share the results. […] No one will even care that you are taking a test” (IDI 21, Female, <25, Not Tested)	Enablement Environmental Restructuring	Goals and Planning Repetition Antecedent
Automatic Motivation	Feelings about testing services & self-testing Misconceptions and assumptions about testing & results	“I felt well because of how the lady treated me that made me open up and talk. These guys are reliable and you are sure about them” (IDI 3, Male, <25, Tested)	“If they find themselves positive, they think there is no one who can guide them, and commit suicide” (IDI 1, Female, <25, Not Tested)	Enablement Modeling	Social Support Self-belief Associations
Reflective Motivation	Agency in deciding what kind of test to pursue and how/when/why Education and discussions with providers to overcome perceived barriers	“Us men are hard to convince to get tested because we think we are well and is not the case. When you go to the hospital, you find someone to teach you and get you tested” (IDI 12, Male, >25, Tested)	“I would have used it with the service provider so that he can show me how to use it because am not a doctor I can do something stupid then it gives me a wrong result or it fails to work” (IDI 20, Male, >25, Not Tested)	Education Persuasion	Shaping Knowledge Antecedent Associations

## Data Availability

The data presented in this study are available on request from the corresponding author. Full data are not publicly available due to privacy restrictions.

## Abbreviations

HTS: HIV Testing Services
EDs: Emergency Departments
HIVST: HIV Self-Testing
KNH: Kenyatta National Hospital
COM-B: Capability-Opportunity-Motivation Behavioral Model
PLHIV: People Living with HIV
IDIs: In-Depth Interviews
VCT: Voluntary Counseling and Testing
